# Bidirectional crosstalk between the peripheral nervous system and lymphoid tissues/organs

**DOI:** 10.3389/fimmu.2023.1254054

**Published:** 2023-09-12

**Authors:** Angela Boahen, Dailun Hu, Murray J. Adams, Philip K. Nicholls, Wayne K. Greene, Bin Ma

**Affiliations:** ^1^ Department of Medical Microbiology, Faculty of Medicine and Health Sciences, Universiti Putra Malaysia, Seri-Kembangan, Selangor, Malaysia; ^2^ Department of Pathogenic Biology, Hebei Medical University, Shijiazhuang, Hebei, China; ^3^ School of Medical, Molecular and Forensic Sciences, Murdoch University, Murdoch, WA, Australia

**Keywords:** neuroimmunology, lymphoid tissues/organs, peripheral nervous system, innervation, neuroimmune cell units, autonomic nervous system

## Abstract

The central nervous system (CNS) influences the immune system generally by regulating the systemic concentration of humoral substances (e.g., cortisol and epinephrine), whereas the peripheral nervous system (PNS) communicates specifically with the immune system according to local interactions/connections. An imbalance between the components of the PNS might contribute to pathogenesis and the further development of certain diseases. In this review, we have explored the “thread” (hardwiring) of the connections between the immune system (e.g., primary/secondary/tertiary lymphoid tissues/organs) and PNS (e.g., sensory, sympathetic, parasympathetic, and enteric nervous systems (ENS)) in health and disease *in vitro* and *in vivo*. Neuroimmune cell units provide an anatomical and physiological basis for bidirectional crosstalk between the PNS and the immune system in peripheral tissues, including lymphoid tissues and organs. These neuroimmune interactions/modulation studies might greatly contribute to a better understanding of the mechanisms through which the PNS possibly affects cellular and humoral-mediated immune responses or vice versa in health and diseases. Physical, chemical, pharmacological, and other manipulations of these neuroimmune interactions should bring about the development of practical therapeutic applications for certain neurological, neuroimmunological, infectious, inflammatory, and immunological disorders/diseases.

## 1 Introduction

### 1.1 Central nervous system and peripheral nervous system

The nervous system comprises the central nervous system (CNS) and the peripheral nervous system (PNS). The CNS, including the brain and spinal cord, is responsible for integrating, coordinating, and maintaining the overall function and well-being of the human body (1). In association with social activities, the human cerebral cortex has become rapidly developed and continuously improved, producing advanced functional activities such as language, thinking, learning, and memory. Therefore, mammals are able not only to adapt to changes in the environment, but also to recognize and actively transform the environment. In PNS, bundles of nerve fibers (axons and dendrites projecting from neuron cell bodies located in the spinal cord and ganglia) and their nerve terminals are surrounded by connective tissue layers and are organized into nerves. A branching network of intersecting nerves then forms a nerve trunk and nerve plexus. Functionally, the PNS consists of somatic afferent (somatic sensory), somatic efferent (somatic motoric), visceral efferent (autonomic; including sympathetic and parasympathetic), and visceral afferent (visceral sensory) nerve fibers (2). The PNS relays sensory information to the CNS and transmits motor commands to regulate bodily functions and facilitate interactions with the external environment (1, 3, 4). In the PNS, the autonomic nervous system (ANS) is one of the main biological systems that regulates involuntary physiologic processes including heart rate, blood pressure, respiration, digestion, and sexual arousal (5).

The ANS has three anatomically distinct divisions: sympathetic, parasympathetic, and enteric (5). The sympathetic nervous system (SNS) and parasympathetic nervous system (PSNS) work together to maintain homeostasis and to regulate various bodily functions. Whereas the SNS is responsible for the “fight-or-flight” response, the PSNS is often referred to as the “rest and digest” system (6). The SNS emanates from the lateral horns of the gray matter of the entire thoracic and upper three lumbar cords of the spinal cord. It uses short communication branches (preganglionic fibers) to connect with the sympathetic trunks on both sides of the spinal cord and then sends out postganglionic fibers (from the sympathetic trunk nerves) to control the activities of the organs and blood vessels in the chest and abdomen (7). The SNS plays a crucial role in preparing the body for rapid responses in stressful or threatening situations, allowing individuals to deal effectively with challenges or dangers (6). The PSNS arises from the brain stem (midbrain, pons, and medulla oblongata) and sacral regions of the spinal cord. Its preganglionic fibers exchange neurons in the parasympathetic ganglion, from which postganglionic fibers emanate to smooth muscle, cardiac muscle, and glands (8). The PSNS promotes relaxation, conserves energy, and facilitates normal bodily functions such as digesting food, slowing the heart rate, and promoting rest/recovery (6).

The vagus nerve (cranial nerve X) is a long and complex nerve that originates in the brainstem (dorsal motor nucleus of the vagus nerve, nucleus ambiguus, and the solitary nucleus) and extends throughout the body (9). It travels down the neck, alongside the esophagus, and branches out to innervate the heart, lungs, stomach, liver, pancreas, intestines, and other organs. It is involved in a wide range of bodily functions (including the regulation of the heart rate, the control of respiratory muscles, and the stimulation of digestion) and exerts an influence on gut function/motility (10). The vagus nerve contains 20% “efferent” (motor) fibers and 80% “afferent” (sensory) fibers (11). The sensory fibers of the vagus nerve, most of which are non-myelinated and slow-conducting C-fibers, provide information/sensation about the state/functioning of, for example, the heart, lungs, gastrointestinal tract (GIT), and other organs/structures in the thoracic cavity and abdomen. These sensory signals can help the brain monitor and regulate body functions (8). For example, the vagus nerve carries information about physical changes (e.g., blood pressure changes, stretching of the gut) and chemical changes (e.g., oxygen levels, secretions of bacteria in the intestine) in the body, allowing the brain to make appropriate adjustments and to maintain homeostasis. Furthermore, the vagus nerve is also connected to various parts of the brain, influencing emotions and memory (8).

### 1.2 Crosstalk between PNS and immune system

The function of the immune system is to recognize and defend the host against pathogens (e.g., bacteria, viruses, and fungi) and substances or cells that appear foreign and harmful (12). This system consists of immune organs (e.g., bone marrow, spleen, lymph nodes, tonsils, Peyer’s patches, cecum/appendix, thymus), immune cells (lymphocytes, mononuclear phagocytes, neutrophils, basophils, eosinophils, mast cells, and platelets), and immune active substances (e.g., antibodies, lysozymes, complement, immunoglobulin, interferon, interleukin, tumor necrosis factor (TNF), and other cytokines) (12). It is divided into innate immunity (non-specific immunity) and adaptive immunity (specific immunity), of which adaptive immunity is further divided into humoral immunity and cellular immunity (12–14). Interestingly, in addition to the immune cells, some other cells (e.g., mesenchymal stromal/stem cells, fibroblasts, endothelial cells, osteoblasts, neurons, and Schwann cells) can exhibit significant immune functions (e.g., by secreting cytokines/chemokines/growth factors, promoting inflammation, antigen presentation, immunosuppression, and antimicrobial effects) under certain conditions (e.g., infection and inflammation) (15–19).

The nervous system regulates and controls the functions of the body’s organs and systems. However, instead of being isolated, our body systems are interconnected and dependent on each other. For example, the nervous system and immune system can mount a variety of essential coordinated responses to danger (20–23). Usually, the CNS influences the immune system in a general fashion by regulating the systemic concentration of humoral substances (e.g., cortisol and epinephrine) (24). The psychosocial state of a person can have a direct impact on his/her immune system. Stimuli such as over-eating, sleep, stress, and even operant conditioning in which a positive or negative stimulus is paired with a particular behavioral outcome, can influence the immune response significantly (25). For example, overeating or obesity can lead to reduced immunity, as excessive fat accumulation and altered metabolic processes associated with obesity can negatively impact immune system function, potentially increasing susceptibility to infections and impairing immune responses (26). Adequate sleep can boost immunity, as it allows the immune system to function optimally by promoting the production of immune cells and the release of cytokines that help defend the body against infections and diseases (27). Stress can potentially weaken innate and adaptive immunity to infections such as coronavirus disease 2019 (COVID-19), as chronic stress seems to suppress immune responses, making individuals more susceptible to infections, including the virus causing COVID-19 (28, 29).

Another example of neuroimmune interaction is that psychological stress/depression can worsen asthmatic symptoms (30). In addition, neuroendocrine hormones such as corticotropin-releasing factor, leptin, and alpha-melanocyte-stimulating hormone can regulate cytokine secretion and balance (31). Furthermore, the immune response can have surprising effects on brain activities, including body temperature, sleep, and feeding behavior when it detects injury/damage or infection/inflammation (32). For example, the well-known feeling of sleepiness and the lack of appetite associated with fever is, in part, a result of proinflammatory mediators (e.g.,interleukin-1 (IL-1), tumor necrosis factor-α (TNF-α), interleukin-6 (IL-6), and prostaglandins) acting on the hypothalamus (33).

The PNS communicates specifically with the immune system according to local interactions/conditions (34, 35). For example, the main immune organs (e.g., bone marrow, thymus, spleen, and lymph nodes) are supplied with an autonomic efferent (mainly sympathetic) innervation and afferent sensory innervation, and both classic (catecholamines and acetylcholine (ACh)) and peptide neurotransmitters are probably involved in this type of neuroimmune modulation (36–38). However, despite these above-mentioned studies that indicate the occurrence of functional interconnections between the immune and nervous systems, data available on the mechanisms of this bidirectional crosstalk between the PNS and immune system are frequently incomplete and not always focused on their relevance for neuroimmune modulations in neuroimmunological, infectious, immunological disorders/diseases. The goal of this review is to explore the “thread” (hardwiring) of the connections between the immune system (e.g., primary/secondary/tertiary lymphoid tissues/organs) and PNS (e.g., sensory, SNS, PSNS, and enteric nervous systems (ENS)) in health and disease *in vitro* and *in vivo*.

## 2 Neuroimmune interactions in primary lymphoid organs in health and diseases

### 2.1 Neuroimmune interactions in the thymus

The thymus is a vital primary lymphoid organ responsible for the differentiation/maturation of bone-marrow-derived T cell precursors (thymocytes) and their subsequent migration to T-cell-dependent areas in peripheral secondary lymphoid tissues/organs (e.g., spleen, lymph node, and mucosa-associated lymphoid tissue (MALT)). Positive selection occurs in the cortex in which thymocytes with low reactivity to the major histocompatibility complex (MHC) are deleted/eliminated, whereas negative selection takes place in the medulla in which cells that show reactivity against self-antigens are eliminated. (39). These intricate selection processes rely on the precise organization and compartmentalization of parenchymal cells facilitating interactions and signaling pathways inside the thymus (39). The thymic microenvironment includes nerves/nerve fibers, which are believed to regulate T cell development and thymic endocrine function by influencing the secretion of self-hormones such as thymulin, thymopoietin, and thymosins (40, 41).

Macroscopically, nerve fibers can be seen to originate from postganglionic cell bodies (found in the superior cervical and stellate ganglion of the sympathetic chain) and provide sympathetic innervation to the human thymus (42). In addition, the thymus is innervated by acetylcholinesterase (AChE)^+^ nerve fibers from the vagus nerves (including the recurrent laryngeal nerves) and phrenic nerves (43). Microscopically, a variety of experimental approaches (e.g., histochemical, immunohistochemical, virus/dye tracing) have revealed thymic sensory, sympathetic, and parasympathetic innervation systems in several animal species (e.g., mouse, rat, rabbit, and human) (37, 41, 44–48).

The sympathetic innervation influences the development, migration, and homing of lymphocytes within the thymus. It modulates the expression of adhesion molecules (e.g., Thy-1 (CD90)) on thymic epithelial cells (TECs), which facilitate the interaction and movement of developing T cells (49). This intricate interplay between sympathetic fibers and thymic cells helps to orchestrate the maturation processes and the formation of a diverse and functional T-cell repertoire (50). For example, chronic stress-induced loss of sympathetic innervation can affect thymic development and T cell development/maturation (51). The parasympathetic fibers release ACh, which acts on muscarinic acetylcholine receptors (mAChRs) present on the immune/non-immune cells inside the thymus (52). However, the specific roles/functions of the parasympathetic innervation on thymic physiology/pathophysiology are not as well understood compared with those of the sympathetic innervation.

Several neuropeptides (e.g., neuropeptide Y (NPY), vasoactive intestinal polypeptide (VIP), substance P (SP), and calcitonin gene-related peptide (CGRP)) have been shown to contribute to the regulation/modulation of immune processes (e.g., T cell development and maturation) inside the thymus (41, 50). For example, SP and CGRP play essential roles in the differentiation, proliferation, and maturation of thymocytes. CGRP can inhibit the proliferation of virgin mature T cells inside the thymus (53). In addition, SP and CGRP might also be involved in the promotion of T cell apoptosis inside the thymus (53). T cell development and function are also regulated by other neuropeptides. For example, VIP can impact three crucial thymocyte functions, namely cytokine production, migration/mobility, and apoptosis (53, 54).

Previous studies have shown that thymic innervation is mainly associated with the blood vessels inside the thymus, and only sparse nerve fibers are present in the cortex, cortex-medulla junction, and medulla (37, 41, 44–47). However, our studies have demonstrated, by means of a general neuronal marker (neurofilament heavy chain (NF-H)) and the non-myelinating Schwann cellmarker (NMSC)-glial fibrillary acidic protein (GFAP), that all components of the thymus including the capsule, cortex, cortex-medulla junction, and medulla are extensively innervated (46, 55).

Several neuronal/glial cell markers have been utilized to identify the types of nerve fibers inside the thymus and other lymphoid tissue/organs (**Table 1**). Normally, protein gene product 9.5 (PGP9.5), NF-H, βIII tubulin, and microtubule-associated protein 2 (MAP2) are used as general neuronal markers for the characterization of nerve fibers/nerves/neurons/neuroendocrine cells inside the lymphoid organs. Tyrosine hydroxylase (TH) and β2-adrenergic receptor (β2-AR) have been used for the characterization of sympathetic nerve fibers/nerves/neurons, whereas choline acetyltransferase (ChAT) and vesicular acetylcholine transporter (VAChT) have been employed as markers for the parasympathetic cholinergic fibers inside the lymphoid organs. In addition, transient receptor potential vanilloid 1 (TRPV1) and CGRP have been used for the identification of visceral/somatic sensory nerve fibers inside the lymphoid tissues and organs. In addition to the neuron and nerve fibers expressing neurotransmitters/neuropeptides and their receptors, other cells (including immune cells) or structures (including reticular tissue, blood/lymphatic vessels) inside the lymphoid organs might express these markers (**Table 2**). The immune cells within the thymus express various receptors for neurotransmitters/neuropeptides, allowing them to sense and respond to neuronal signals. For example, thymocytes possess β2-AR, cholinergic receptors, and other neurotransmitter/neuropeptide receptors that can influence thymocyte activation, proliferation, and migration inside the thymus (49, 50).

**Table 1:** Neuronal/glial cell markers used to study the innervation of lymphoid tissues/organs.

Markers (histochemistry, immunohistochemistry, or transgenic)	Neuron/nerves/glial cells	Lymphoid tissues/organs
Protein gene product (PGP9.5)	Pan-neuronal markers Neuroendocrine cells	Spleen/GALT (36, 56–58) Thymus (56, 59) Bone marrow (60) TLT (in melanoma and pancreatic cancer) (61, 62)
Tyrosine hydroxylase (TH)	Sympathetic fibers Dopaminergic fibers	LN (48, 56, 63, 64) Thymus (41, 59, 64, 65) Bone marrow (64, 66–71) Spleen (56, 64, 72)
Acetyl- cholinesterase (AChE)	Parasympathetic fibers Neuromuscular junctions	Thymus (41, 65)
Choline acetyltransferase (ChAT)	Sympathetic fibers Dopaminergic nerve fibers	Thymus (52) Mesenteric LN (73) GALT/spleen (74) LN (63) Bone marrow (66)
Transient receptor potential vanilloid 1 (TRPV1)	Sensory nerve fibers	Inflamed islets of Langerhans (75)
Anti-β2-AR	Sympathetic fibers	Immune cells (47, 49) LN (76)
Calcitonin gene-related peptide (CGRP)	Sensory fibers from DRG Motor fibers from anterior horn of spinal cord	Bone marrow/thymus (37) Bone marrow (67, 69–71) Spleen (67) PP (58)
NF-H Neurofilament heavy chain (NFH); Neurofilament 200 (NF-200)	Pan-neuronal markers	Spleen (77) LN/PP/thymus/spleen (58, 78–80) Thymus (46, 59)
Vesicular acetylcholine transporter (VAChT)	Cholinergic neurons/nerves Nerve terminals	Bone marrow (66) Thymus/spleen/LN/PP (56)
β III-Tubulin	Pan-neuronal marker	LN (63) Spleen (67) Bone marrow (67, 69) TLT in colon (81)
Microtubule-associated protein 2 (MAP2)	Pan-neuronal marker	Bone marrow (68)
Glial fibrillary acidic protein (GFAP)	NMSCs associated with C-nerve fibers (sensory/efferent), postganglionic sympathetic fibers, some of the preganglionic sympathetic/parasympathetic fibers, and the motor nerve terminals at neuromuscular junctions	Thymus (55) Mesenteric LN (82) Spleen (83) PP (80, 84) Bone marrow (85)

GALT, gut-associated lymphoid tissue; LN, lymph node; PP, Peyer′s patches; TLT, tertiary lymphoid tissue.

**Table 2:** Expression of neurotransmitters/neuropeptides and their receptors in lymphoid tissues and organs.

Markers	Location and Functions in PNS	Lymphoid tissues/organs
Noradrenaline (Norepinephrine; NE)	Sympathetic nerve fibers	Bone marrow (cells and nerve fibers) (37) Spleen and LN (lymphocytes and macrophages) (37)
Muscarinic acetylcholine receptor	Main end-receptor stimulated by ACh; released from postganglionic fibers	GALT (DC and nerve fibers) (36) Spleen/LN (cells and nerve fibers) (37)
Substance P (SP)	Neurotransmitter and a modulator of pain perception; proinflammatory	Thymus (pain fibers) (41) Bone marrow and thymus (thymocytes) (37) Spleen and LN (lymphocytes and macrophages) (37) Bone marrow (66) PP (58)
Vasoactive intestinal polypeptide (VIP)	Neuromodulator and neurotransmitter; anti-inflammatory	Thymus (pain fibers) (41) Bone marrow (37, 66, 68, 86) PP (58)
Neuropeptide Y (NPY)	Neurotransmitter during cellular communication in GABAergic neurons; proinflammatory	Bone marrow (32, 66), Thymus (thymocytes) (32) Spleen (72)
Dopamine	Transmitter for movement, memory, pleasurable reward, and motivation	Thymus (thymocytes) and spleen/LN (32)
Acetylcholine (Ach)	Excitatory neurotransmitter; primary neurotransmitter for parasympathetic nerve fibers; somatic nervous system (neuromuscular junction)	Thymus (thymocytes, TECs, and nerve fibers) (32) Spleen/LN (T cells and nerve fibers) (32)
Glutamate	Excitatory neurotransmitter	Bone marrow (68)

DC, dendritic cell; GALT, gut-associated lymphoid tissue; LN, lymph node; PP, Peyer′s patches; TECs, thymic epithelial cells.

Numerous studies have demonstrated the direct interaction between nerve fibers and several types of cells inside the thymus. For example, T cells, TECs, and mast cells have been revealed to be closely associated with sympathetic nerve fibers inside the thymus (49, 87). In addition, Wülfing et al. have shown that the NF-H^+^ nerve fibers are intimately associated with the antigen-presenting cells (APCs; demonstrated by major histocompatibility complex II (MHC II) immunostaining) in the thymus (78). We have observed the close proximity of nerve fibers to various subsets of thymocytes (e.g., CD4^+^, CD8^+^, and CD4^+^CD8^+^), dendritic cells (DCs; e.g., B220^+^, CD4^+^, CD8^+^ and F4/80^+^), macrophages (Mac1^+^ and F4/80^+^), and B cells strongly indicating that this innervation affects both T cell development and APC antigen-presentation/cytokine secretion inside the mouse thymus (46).

In another of our studies, we utilized GFAP to characterize the NMSCs inside the mouse thymus (55). The extensive GFAP staining in all compartments of thymus indirectly demonstrated the Group C nerve fibers (sensory/efferent), the postganglionic sympathetic fibers, some of the preganglionic sympathetic/parasympathetic fibers, and the motor nerve terminals at neuromuscular junctions (55). We also observed close “synapse-like” association of NMSC processes with various subsets of DCs (e.g., B220^+^, CD4^+^, and CD8^+^), and lymphocytes (B cells, CD4^+^/CD8^+^ thymocytes) (55). Since the NMSC can function as professional APCs (88, 89), their interactions with immune cells indicate their potential immune regulation function inside the thymus and other primary/secondary lymphoid tissue/organs.

### 2.2 Neuroimmune interactions in bone marrow

Bone marrow, a component of bone, not only engages in blood cell production, but might have functions in immune responses (90, 91). For example, as the major source of immune cells, the bone marrow is an essential target and actor in infection (92). The bone marrow microenvironment, also known as the hematopoietic niche, is a complex network of cells, extracellular matrix, and signaling molecules that support the development, differentiation, and maintenance of hematopoietic stem cells (HSCs). It provides a specialized niche in which stem cells interact with their surrounding environment for self-renewal and lineage-specific differentiation (91, 92).

Because of the various locations of bones, their innervation varies regionally, with the different bones and bone regions/components exhibiting variations in their nerve density and distribution (93, 94). For example, for the femur (including its bone marrow), the innervation is mainly through the femoral nerve, which consists of somatic sensory/motor nerve fibers and sympathetic/parasympathetic nerve fibers (70). The complex and dynamic innervation of the bone marrow plays a crucial role in regulating hematopoiesis, immune responses (e.g., the homing of memory T/B cells), and bone metabolism (60, 90, 95–97). Normally, the bone marrow contains two types of nerve fibers, namely sensory nerve fibers (4%) and autonomic nerve fibers (96%) (66). Sensory nerve fibers innervate the bone marrow and transmit various types of sensory information, including pain, temperature, and pressure (93). These sensory nerves can detect changes in the microenvironment of the bone marrow and relay this information to the CNS. Therefore, sensory innervation is critical for the detection of potential injury/damage, inflammation, or other pathological conditions within the bone marrow (66, 96).

Regional autonomic nerves, including sympathetic and parasympathetic nerves, also innervate the bone marrow. The autonomic nerves play a crucial role in regulating various physiological processes of bone marrow (66). For example, sympathetic nerves release norepinephrine (NE), which can modulate hematopoiesis by influencing HSC activities (e.g., quiescence, proliferation, and mobilization) and the bone marrow microenvironment (96). Certain conditions, such as aging and stress, can reduce the number and size of nerve fibers inside the bone marrow (69). In addition, denervation or a reduction in sympathetic nerve fibers might lead to a reduction/loss of HSCs (85). The parasympathetic nerves, on the other hand, might have a regulatory role in bone metabolism/remodeling through the dual effects of cholinergic signals on osteoclasts (apoptosis) and osteoblast function (proliferation and bone formation) (66, 98). However, their presence inside the bone marrow is still controversial, and their exact roles remain unclear (96).

Several markers have been utilized to detect the nerve fiber/neurons inside bone marrow (**Table 1**). TH has been used for the characterization of sympathetic nerve fibers/neurons, whereas ChAT and VAChT have been utilized for locating parasympathetic cholinergic fibers inside the bone marrow. According to previous studies, mixed nerve fibers (myelinated and non-myelinated) enter the medullary cavity through the nutrient foramen or Haversian canals (99). After entering the bone marrow, most nerve fibers accompany the blood vessels, whereas some do not (67). The nerves of the bone marrow communicate and interact with immune cells, including hematopoietic cells, lymphocytes, and myeloid cells. This type of interaction occurs through a few mechanisms, including the release of neurotransmitters/neuropeptides (e.g., SP, CGRP, NPY) from nerve fibers and the expression of receptors for these signaling molecules on immune cells (**Table 2**). These molecules (neurotransmitters/neuropeptides and their receptors) in HSCs have been shown to regulate hematopoiesis, immune cell functions/activities, the bone marrow microenvironment, and bone metabolism (67, 96, 100, 101). For example, NPY receptors (e.g., Y1, Y2, Y4, and Y5) are highly expressed in HSCs, and NPY is essential for the mobilization and proliferation of HSCs inside the bone marrow (102).

The mechanisms of bidirectional crosstalk between the PNS and bone marrow can be investigated by examining the local interactions between nerve fibers and other cell populations inside the bone marrow. For example, immunohistochemical and immuno-electron microscope (EM) studies have shown that the nerve fibers/endings have close contact with hematopoietic cells and osteoblasts inside the bone marrow (68). In addition, nerves in the bone marrow also interact with stromal cells, which include osteoblasts, osteoclasts, mesenchymal stem cells, and endothelial cells. These local interactions are crucial for regulating bone remodeling, hematopoiesis, and the maintenance of the bone marrow microenvironment (67, 68, 103). These interesting studies have revealed important elements of anatomical and physiological bidirectional crosstalk between the nerve fibers and parenchyma/stromal components of bone marrow.

## 3 Neuroimmune interactions in secondary lymphoid tissue/organs

### 3.1 Neuroimmune interactions in lymph node

Lymph nodes function as filters for harmful substances/waste products and meeting points for various immune cells. Their highly organized structure enables efficient immune responses, helping to defend the body against pathogens/foreign substances/tumors and to maintain overall health (104). The regional distribution of lymph nodes inside the mammalian body results in them receiving regional innervation. The innervation of lymph nodes is complex and can vary depending on factors such as the location, size, and function of the lymph node. Both the sympathetic and parasympathetic branches (from the vagus nerve) of the ANS are involved in this innervation (47, 105). In addition, lymph nodes have a sensory (afferent) innervation since they are responsible for immune responses against injury/infection/tumors in specific regions (63). Nociceptors are specialized nerve endings that detect and transmit signals related to pain and potential tissue injury/damage. They can regulate the immune response inside the lymph node through neuropeptides (e.g., SP and CGRP) and affect the immune cells/stromal cells expressing these peptide receptors (**Table 2**) (105). For example, activation of the innervation of the lymph node can lead to antigen retention in the lymph, whereas the blocking of this neuronal activity can restore antigen flow inside the lymph nodes (106).

Several neuronal/glial markers have been used to identify the types of nerve fibers inside lymph nodes (**Table 1**). For example, TH and anti-β2-AR have been utilized to characterize the sympathetic nerve fibers/neurons, whereas ChAT and VAChT have been employed as markers for the parasympathetic cholinergic fibers inside the lymph node (**Table 1**). The sympathetic and sensory innervation of lymph node has been revealed by using these markers in numerous studies. Nerve fibers have been demonstrated to enter the lymph node from the hilum, to accompany the blood vessels, to pass through the medullary region (with fibers entering the medullary cord and sinus), and to form subscapular plexuses. From these plexuses, nerve fibers enter the cortical and paracortical areas (105). However, these studies have a few limitations. The first is that only sparse neurons/nerve fibers/nerve endings have been identified in some investigations (44, 48). Second, even though the major nerves (after entering the lymph node through hilum) have been shown in some studies, the fine nerve fibers inside the lymph node have not (56, 63). Third, the innervation of certain regions (e.g., B cell follicles and their germinal centers) has not been revealed (105). Fourth, the potential parasympathetic innervation (56) has not been demonstrated in some studies (105). Lastly, the close interaction/contact between nerve fibers/nerves and immune cell/non-immune cells (48) has not been well documented in some studies (56).

In our previous studies, NF-H and GFAP were utilized to investigate the innervation of mouse lymph nodes (82). We demonstrated the presence of extensive nerve fibers in all compartments (including B cell follicles) of mouse lymph node, and some nerve fibers had close contacts/associations with blood vessels (including high endothelial venules (HEVs)) and lymphatic vessels/sinuses. We also showed the close contacts/associations between nerve fibers and immune cells (e.g., various subsets of DCs (e.g., B220^+^CD11c^+^, CD4^+^CD11c^+^, CD8a^+^CD11c^+^, and Mac1^+^CD11c^+^), Mac1^+^ macrophages, and B/T lymphocytes). However, one limitation of our study was that we did not identify the types of nerve fibers inside the lymph node because of the use of general neuronal markers. This type of nerve-immune interaction (demonstrated by high-resolution microscopic imaging) inside the lymph node has however been observed in studies by other investigators (48, 105, 107). In our studies, we also used GFAP for the identification of NMSCs inside the lymph node (82). We observed extensive immunostaining of NMSCs in all compartments of mouse lymph node. In addition, we found NMSC processes interacting with various subsets of DCs (e.g., CD4^+^CD11c^+^, CD8^+^CD11c^+^ DCs), macrophages (F4/80^+^ and CD11b^+^ macrophages), and lymphocytes (82). Since Schwann cells can express major histocompatibility complex II (MHCII) and act as APCs under pathological conditions, their interactions with T cells (e.g., T helper cells) or other immune cells (e.g., macrophage and DC) might lead to T cell/macrophage/DC activation and cytokine release (89, 108).

In addition to light microscopy, EM has been utilized to study nerve-immune cell interactions. For example, nerve fibers have been observed lying in close contact with plasma cells inside the axillary lymph nodes (109, 110). This type of interaction demonstrated by ultrastructural analysis has provided powerful evidence/confirmation for nerve-immune cell interactions inside the lymph node and other lymphoid tissues/organs.

### 3.2 Neuroimmune interactions in the spleen

The spleen is the largest secondary lymphoid organ responsible for the filtration/storage of blood and the immune response against bacterial/viral infection (111). It is enclosed in a capsule of fibrous and elastic tissue that extends into the parenchyma as trabeculae. It is separated into the blood-containing red pulp (primarily for innate immunity) and the lymphoid-cell-containing white pulp (primarily for adaptive immunity) by an interface, namely the marginal zone (part of the white pulp) (111).

Splenic sympathetic innervation arises from the celiac plexus, the left celiac ganglion, whereas parasympathetic innervation comes from the vagus nerve (112). The sympathetic nerves enter the spleen together with blood vessels inside the trabeculae, whereas the parasympathetic nerve fibers can reach the spleen via both of its tips (83, 113). However, the presence of sensory nerve fibers inside the spleen remains controversial (112, 114).

Several neuronal/glial markers have been utilized to identify the types of nerves/nerve fibers inside the spleen (**Table 1**). For example, TH has been used for the characterization of sympathetic nerve fibers/neurons, whereas ChAT and VAChT have been employed for parasympathetic cholinergic fibers inside the spleen (**Table 1**). The peripheral innervation of spleen has been revealed by means of these markers in several studies. The splenic nerves have been shown to enter the spleen at the splenic hilum along the splenic artery, to travel in the plexuses together with the vasculature, to continue into the spleen in the trabeculae with the trabecular plexi, and to extend into the white pulp (including the splenic nodules, marginal zones, and periarteriolar lymphoid sheaths (PALS)) (77, 112). However, a few limitations to the above studies should be mentioned. The first is that, in some investigations, only sparse neurons/nerve fibers/nerve endings have been identified in the spleen (59, 72). Second, even though the major nerves (after entering the spleen through hilum) have been shown, some studies have not identified fine nerve fibers inside the spleen (64). Third, the innervation of certain regions (e.g., B cell follicles and their Germinal centers) has often not been established (64). Fourth, the potential parasympathetic innervation (64, 73) has not been shown in some of the studies. Lastly, the local close contact/interactions between nerve fibers and immune cell/non-immune cells have sometimes not been well documented (78, 79).

In our previous studies, we used NF-H and GFAP to investigate murine splenic innervation (77, 83). We demonstrated the presence of extensive nerve fibers in all splenic compartments (including the splenic nodules, PALS, marginal zones, trabeculae, and red pulp) and close associations between these nerve fibers with blood vessels (including central arteries, marginal sinuses, penicillar arterioles, and splenic sinuses) (77). In addition, we observed close associations between nerve fibers and various subsets of DCs (CD11c^+^), macrophages (Mac1^+^ and F4/80^+^), and lymphocytes (B cells, T helper cells, and cytotoxic T cells) (77). However, one limitation of our study was that, by using NF-H as a general neuronal marker, we did not identify the types of nerve fibers inside the spleen. This type of nerve-immune interaction (demonstrated by high-resolution microscopic imaging) inside the spleen has been observed in some studies by other investigators (72, 114–116). For example, Kirkland et al. have shown the close association of TH^+^ sympathetic fibers with CD3^+^ T cells in the human and porcine spleen (72). In addition, Murray et al. have also demonstrated close associations between TH^+^ (sympathetic)/ChAT^+^ (parasympathetic) nerve fibers with CD3^+^ T cells in the murine spleen (73).

These above-mentioned studies concerning splenic innervation and nerve-immune cell communication enrich our knowledge of the effects of the PNS on the cellular- and humoral-mediated immune responses in healthy and infectious/non-infectious conditions. For example, vagus nerve stimulation (anterograde efferent fiber stimulation and anterograde afferent fiber stimulation) seems to activate neuroimmune circuits (e.g., C1 neurons (in the brain stem)- SNS-splenic nerve-spleen-kidney axis for anterograde afferent fiber stimulation) inside the spleen and protect mice from kidney from ischemia-reperfusion injury (117). On the contrary, infection/inflammatory conditions might also affect splenic innervation. For example, sepsis can lead to the loss of noradrenergic (sympathetic) nerves inside the human spleen possibly because of altered immune responses (e.g., increased inflammatory cytokines, immunosuppression, or reduction of nerve growth factor (NGF) from immune cells) (115). In addition, Kelley et al. have shown that murine acquired immunodeficiency syndrome (AIDS) can lead to splenic sympathetic nerve destruction (118).

### 3.3 Neuroimmune interactions in GALT

MALT refers to a component of the immune system found in various mucosal surfaces throughout the body (119). Specific MALT structures include the tonsils, adenoids, Peyer’s patches in the small intestine, and lymphoid follicles/tissues in the respiratory/urogenital mucosa. The primary function of MALT tissue is to protect mucosal surfaces from invading pathogens/foreign substances and to trigger immune responses against them (119). Another function of MALT is immune tolerance at various mucosal surfaces (120). GALT is a specific MALT component located in GIT. It includes structures such as Peyer’s patches, scattered immune cells, diffuse lymphoid tissues, and aggregated lymphoid tissues (e.g., in the cecum, colon, and appendix) (121, 122). It plays a vital role in immune surveillance/tolerance, in defense against pathogens, and in maintaining gut homeostasis (122).

The neural regulation of GIT function relies on a delicate balance of intrinsic and extrinsic nervous divisions (123). The ENS, which forms the intrinsic division of the GIT nervous system, consists of intrinsic primary afferent neurons, interneurons, and motor neurons located within the myenteric plexus (Auerbach’s plexus) and the submucosal plexus (Meissner’s plexus) (124). It might operate independently of the brain/spinal cord but relies on innervation from the extrinsic nervous division, which consists of nerve branches from the vagus nerve (containing visceral sensory and parasympathetic fibers), the sympathetic trunk/ganglion, and the dorsal root ganglion (DRG; containing visceral sensory neurons) (124).

The GIT nervous system communicates bidirectionally with the immune system through various mechanisms (125, 126). It can release neuropeptides/neurotransmitters/cytokines that modulate immune cell function, such as promoting the release of proinflammatory cytokines or suppressing immune responses (127–129). For example, in GIT, VIP has been shown to modulate the recruitment of intestinal group 3 innate lymphoid cells (through VIP receptor (VPAC)) and the formation of postnatal intestinal lymphoid tissues, thereby providing protection against enteric pathogens (independent of the gut microbiota or adaptive immunity) (130). The CGRP-containing afferent nerve fibers (capsaicin-sensitive) can have protective anti-inflammatory effects and reduce mucosal damage through neuropeptides (primarily CGRP) released from their peripheral endings (127).

Immune cells in GALT can also produce cytokines/neurotransmitters and other signaling molecules that can influence ENS activity (131, 132). For example, enteric neurons can trigger the activation/degranulation of mast cells through neuropeptides (e.g., VIP)/hormones, and vice versa, mast cells can affect the function of enteric neurons through neurotransmitters (e.g., serotonin and histamine)/tryptase (132). Alterations in ENS function and neurotransmitter signaling have been observed in patients with inflammatory bowel disease (IBD) and inflammatory bowel syndrome (IBS). Manipulation of the GIT nervous system (e.g., vagus stimulation) can be utilized for certain diseases such as IBD (131, 133, 134). However, vagus stimulation may also lead to severe complications such as gastric ulcers, since an increased vagal parasympathetic tone has been associated with peptic ulcer formation (135).

The sympathetic nerve fibers have close associations with immune/non-immune cells inside the GALT (e.g., isolated/aggregated lymphoid follicles and Peyer’s patches), whereas the parasympathetic nerve fibers (from the vagus and sacral spinal nerves S2-S4) extensively innervate the gut wall (up to the myenteric plexus) (123, 131).

Various neuronal markers have been utilized to identify the types of nerve fibers inside the GALT (**Table 1**). For example, TH has been used to characterize the sympathetic nerve fibers/neurons, whereas ChAT and VAChT have been employed for parasympathetic cholinergic fibers inside the GALT (**Table 1**). These markers have revealed the ANS innervation of GALT in numerous studies. We utilized PGP9.5, GFAP, and NF-H to study the GALT innervation (36, 84). For example, in the villus of mouse small intestine, we observed close contacts between PGP9.5^+^ nerve fibers/neurons and immune cells (e.g., B/T cells and DCs) (36). In the Peyer′s patches, we found close associations between NMSC processes (demonstrated by GFAP staining) with B cells by using immunostaining and three-dimensional (3D) confocal microscopy (36). Compared with another study showing only sparse nerve fibers (57), we demonstrated an extensive meshwork of NF-H^+^ presumptive nerve fibers in all compartments of the Peyer′s patches (e.g., lymphoid nodules, interfollicular regions, follicle-associated epithelium, and subepithelial dome) and close associations between some nerve fibers with the blood vessels including HEVs, indicating the neural regulation of blood flow and immune cell dynamics inside the Peyer′s patches. In addition, we also observed close contacts between nerve fibers/endings and B/T cells and various subsets of DCs (e.g., B220^-^, B220^+^, CD4^-^, CD4^+^, CD8^-^, and CD8^+^). However, one limitation of our study was that, by using NF-H as a general neuronal marker, we did not identify the types of nerves/nerve fibers inside the GALT. This type of nerve fiber/immune cell contact has been also reported in some other studies. For example, Vulchanova et al. have shown the close associations of SP^+^ nerve fibers and T cell (CD3^+^)/plasma cells (IgA^+^)/APC (MHCII^+^) inside porcine villus (58).

In addition to light microscopy, EM has been utilized in the study of nerve-immune cell interactions inside the GALT. For example, somatostatin-positive nerve fibers/terminals have been shown to lie in close contact with lymphocytes/plasma cells inside the Peyer′s patches of the cat (136). The synaptic cleft is about 20-220 nm, which is similar to clefts of classical synapses (136).

Of note, this type of close contact between nerve fibers and the immune cells in Peyer′s patches and other secondary lymphoid tissues/organs is a potential route for prion transmission. Since B/T lymphocytes, DCs, natural killer T cells, and macrophages might contain/transport prions, this type of nerve-immune cell contact/association might be responsible for prion transmission (through membrane-membrane contact or via exosomes) from lymphoid tissues to distal PNS/CNS (80, 137, 138).

## 4 Neuroimmune interactions in tertiary lymphoid tissue

### 4.1 Tertiary lymphoid tissue

Tertiary lymphoid tissues (TLTs) are organized lymphoid structures (resembling secondary lymphoid tissue/organs) that develop at sites of chronic inflammation or infection outside of the traditional lymphoid tissues/organs (e.g., lymph nodes and spleen) (139, 140). TLTs often form in perivascular areas in response to disturbed tissue homeostasis in various tissues/organs, including the lungs, liver, brain, salivary glands, and GIT. Since the unencapsulated structure of TLTs allows direct exposure to diverse factors/cells from an inflamed environment, they can promote adaptive immunity under certain pathological conditions (140).

TLTs in tumor tissues share similarities with secondary lymphoid tissues/organs, such as lymph nodes, in terms of their organization and cellular composition (141, 142). They can contain B/T cells, DCs, plasma cells, macrophages, and other immune cells, together with specialized stromal cells/connective tissues that support their structure (143). TLTs in tumor tissues can exhibit features such as lymphoid follicle-like structures with distinct B/T cell zones, germinal center-like structures, and HEVs (144). The presence of intertumoral TLTs is believed to reflect ongoing immune responses against the tumor. For example, TLTs can serve as sites for immune cell activation, antigen presentation, and lymphocyte trafficking. In addition, TLTs within tumors have been associated with favorable clinical outcomes of certain cancers, indicating a potential role in anti-tumor immunity/therapy (143, 145, 146). However, the exact impact of TLTs on tumor progression/metastasis and patient prognosis can vary depending on the tumor type and context.

TLTs are also sometimes present at sites of chronic inflammation in autoimmune diseases such as systemic lupus erythematosus (SLE) and rheumatoid arthritis. These organized accumulations of B/T cells and other cells (e.g., DCs, plasma cells, and macrophages) can resemble secondary lymphoid tissue and produce autoreactive effector T cells against self-antigens inside the human body (140, 144).

### 4.2 Innervation of TLTs

Although lymphoid organs, such as lymph nodes, are known to receive extensive innervation, the innervation of TLTs is less well-characterized. However, studies have indicated that TLTs can have neural components, including neurons/nerve/nerve fibers, suggesting a potential role of innervation in TLT functions at various locations of chronic inflammation or tumors (61, 62, 81).

The TLT innervation might serve several functions. Neural components within TLTs can modulate immune responses by interacting with immune cells and influencing their activities. Neuropeptides (e.g., VIP and SP) and neurotransmitters (e.g., ACh) released by nerve fibers in TLTs are capable of impacting the recruitment, activation, and migration of immune cells (e.g., B cells, T helper cells, cytotoxic T cells, and macrophages), thereby affecting the overall immune response (e.g., chronic inflammation, autoimmunity, anti-tumor immune responses) within these structures (61, 81, 147, 148). In addition, neural signals from the ANS can influence TLT development and maintenance (81). For example, sympathetic and parasympathetic nerves release neurotransmitters (e.g., epinephrine, ACh, serotonin, and gamma-aminobutyric acid (GABA))/neuropeptides (e.g., NPY, VIP, and SP) that affect the local environment and cellular interactions, potentially promoting the formation and organization of TLTs (61, 81, 149). Furthermore, sensory neurons/nerve fibers in TLTs can also affect their immune responses. For example, elimination of TRPV1^+^ sensory neurons (by using capsaicin, an active component of chili peppers) prevents insulitis and diabetes in diabetes-prone non-obese diabetic (NOD) mice. Therefore, TRPV1^+^ sensory neurons might control β cell stress and islet inflammation in mouse experimental autoimmune diabetes (75). The depletion of TRPV1^+^ sensory nerves (pain fibers) by using resiniferatoxin can hinder the formation of TLTs and impede the development of effective protective immune responses against murine melanoma (61).

Neurotransmitters/neuropeptides such as ACh, GABA, serotonin (5-HT), NE, NPY, and neurotensin, which can be produced by tumor cells and immune cells inside the TLT, might affect cancer progression/metastasis through multiple signaling pathways (149, 150). Neurotransmitters/neuropeptides and cytokines/chemokines that regulate tumor cell migration might provide an effective pharmacological approach for inhibiting cancer invasion/metastasis (151–153).

Notably, the extent of TLT innervation might vary depending on the tissue and the specific context of inflammation/infection. For example, by using confocal microscopy and 3D reconstruction, Veres et al. have shown the close contact of sensory nerve fibers with T cell/DCs in allergic airway inflammation (154). By means of EM, the authors also showed close associations between the DCs and nerve fibers (axons) inside the inflamed airways, providing robust evidence for nerve-immune cell contact/interaction (154, 155).

## 5 Mechanisms of neuroimmune interactions in lymphoid tissues

### 5.1 Neuroimmune cell units

A neurological synapse is a specialized junction that allows communication between neurons or between a neuron and a target cell (e.g., smooth/cardiac/skeletal muscle cell, adipose cell, glandular cell, endocrine cell) (156). It is the fundamental unit of information transfer in the nervous system. Although many types of synapses have been described within the brain, they can be divided into two general classes: electrical synapses and chemical synapses (157). At the synapse, electrical signals known as action potentials trigger the release of chemical neurotransmitters from the presynaptic neuron (158). These neurotransmitters then bind to receptors on the postsynaptic cell, transmitting the signal and enabling the relay of information. Synapses are dynamic structures that can be modified through processes such as synaptic plasticity and play a crucial role in learning, memory, and overall brain functions (159, 160).

The immunological synapse is a specialized junction between an immune cell and its target, such as an APC (161–165). It consists of a central supramolecular activation cluster and a peripheral supramolecular activation cluster (166). At the immunological synapse, immune cell receptors, such as the T cell receptor (TCR), interact with antigens presented by the target cell through MHC, leading to immune cell activation and signaling (165). The synapse enables the precise spatial and temporal control of immune responses, regulating immune cell activation, cytokine release, and cytotoxicity. An understanding of the immunological synapse provides insights into immune cell function, immune regulation, and potential therapeutic strategies (161–165).

Despite acknowledgement of the crosstalk/interaction between the immune system and PNS during the past few decades, only some recent studies have begun to reveal the anatomical/morphological and molecular/physiological basis of this type of local interaction (167). Although local contacts/associations between nerve fiber/neurons and immune cells (or other non-immune cells with certain immunological functions) (36, 167, 168) have several similar features to both the neurological synapse and the immunological synapse, we still cannot call them “neuroimmune synapses”, at this stage because of limited evidence and functional studies.

In previous studies, three types of contact between nerve fibers and immune cells have been documented in lymphoid tissue/organs (36, 46, 48, 77, 84). The first is the nerve-immune cell contact (36, 154). The second is the neuron (soma)-immune cell (e.g., DCs) membrane-membrane contact (36). The third is the contact between the immune cells and fine nerve fibers/nerve terminal (endings) (36, 46, 48).

The nerve fibers in the peripheral tissue/organs (including the lymphoid tissue/organs) are relatively static, whereas the immune cells are flexible and mobile. Therefore, this type of close nerve-immune cell association/contact should be dynamic under physiological and pathological conditions (154, 155, 168). Further studies need to be performed to elucidate this type of local interaction/contact between the PNS and immune cells. First, this type of interaction should be studied further by using high-resolution microscopic imaging and 3D reconstruction. In this case, immunostaining by using whole-mount tissue/organs (or thick tissue sections) coupled with tissue-clearing techniques can be used (46, 169–173). After the immunostaining step, optic sectioning and 3D projection/reconstruction might reveal neuroimmune interactions *in situ*, thereby excluding the possibility of the “random colocalization” of nerve fibers and immune cells (173) and establishing that two structures have actual close contact (not attributable to their merely lying near each other or to the viewpoint/resolution limit of the light microscopy). Second, live cell/tissue/organ/animal imaging (174, 175) should be used to study this type of dynamic interaction between nerve fibers and immune cells. Third, this type of contact needs to be confirmed further in tissue sections/co-culture of neurons/glial cell and immune cells by using EM and immuno-EM as described in a few previous studies (175, 176). Once enough anatomical/morphological evidence of nerve-immune cell interaction has been obtained, further molecular/physiological/functional studies should be conducted to reveal the molecular mechanism of this type of local neuroimmune interaction (20, 177–179).

Neuroimmune cell units, also known as neuroimmune complexes, is the term used to refer to localized cellular interactions between neurons/nerves and immune cells within the nervous system. These units represent specialized structures whereby immune cells and neurons/nerves/nerve fibers/nerve endings (terminals) come into close contact allowing direct communication and coordination between the two systems (20). Neuroimmune cell units typically involve specific immune cells (e.g., microglia, B/T cells, mast cells, DCs, macrophages) interacting with neurons/nerves and other glial cells. These interactions, occurring through the formation of physical contacts or synapse-like connections (36, 175, 180), involve the exchange of signaling molecules, including cytokines, chemokines, neurotransmitters, and other immune modulators. Neuroimmune cell units are thought to play a significant role in regulating neuroinflammatory responses, modulating neuroimmune signaling, and influencing disease progression in various neurological/neurological disorders and cancers. An active area of research in neuroimmunology in recent years has aimed at elucidating the formation, dynamics, and functional consequences of these units (167).

**Figure 1:**
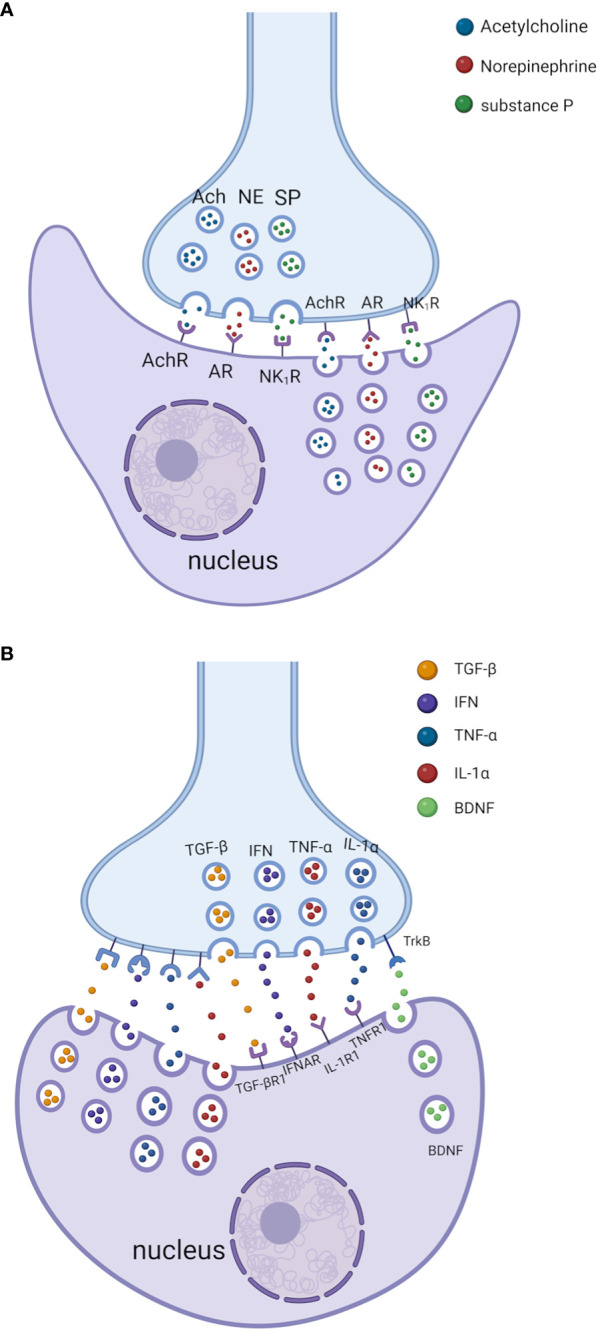
Bidirectional crosstalk between the nerves and immune cells through the neuroimmune cell units. **(A)** Neurotransmitters/neuropeptides (e.g., NE, ACh, and SP) from the neuron/nerve fibers/glial cells of PNS can modulate immune cell functions by binding to specific receptors on immune cells. In addition, neurotransmitters/neuropeptides produced by immune cells can also modulate the PNS function by binding to specific receptors on neuron/nerve fibers/glial cells in PNS. AChR: acetylcholine receptor; AR: adrenergic receptor (for NE); NK1R: neurokinin 1 receptor (for SP). **(B)** Cytokines/chemokines from the immune cells can modulate PNS function by binding specific receptors on neuron/nerve fibers and glial cells in PNS. In addition, cytokines/chemokines produced by the neuron/nerve fibers and glial cells of PNS can also modulate immune cell function by binding to specific cytokine receptors on immune cells. Some immune cells can secrete brain-derived neurotrophic factor (BDNF) that can regulate the activity of nerve fibers by binding with its receptor Tropomyosin receptor kinase B (TrkB). Please note that the space (cleft) between the nerve terminals and immune cells is exaggerated for better presentation. TGF-βR1, transforming growth factor beta receptor 1; IFNAR, interferon-alpha/beta receptor alpha chain; IL-1R1, IL-1 receptor, type 1; TNFR1, tumor necrosis factor receptor 1. The images were created by BioRender.

Several mechanisms might be involved in the formation and functions of the neuroimmune cell units. First, neurotransmitters/neuropeptides (e.g., NE, ACh, SP, and VIP) from the neuron/nerve fibers/glial cells of PNS can modulate immune cell functions by binding to specific receptors on immune cells (**Figure 1A**) (20, 181). Immune cells (e.g., monocytes, macrophages, DCs, and T/B cells) and non-immune cells (e.g., endothelial cells) often express receptors such as muscarinic/nicotinic AChRs and α-/β-AR receptors (36, 182). Activating these receptors by neurotransmitters/neuropeptides can influence immune cell functions, cytokine production, and inflammatory processes, contributing to the neuroimmune crosstalk observed in various physiological and pathological conditions.

The α7 nicotinic acetylcholine receptor (α7nAChR) on macrophages and other immune cells has been reported to mediate the cholinergic anti-inflammatory effects of the vagus nerve (183). Activation of α7nAChR by ACh can promote the phosphorylation and activation of Janus kinase 2 (JAK2), which then promotes the phosphorylation and nuclear entry of signal transducers and activators of transcription 3 (STAT3), reducing the expression of proinflammatory cytokines (184). In addition, activation of α7nAChR can also inhibit the degradation of nuclear factor of kappa light polypeptide gene enhancer in B-cells inhibitor, *alpha* (IκBα) and translocation of nuclear factor kappa B (NF-κB), therefore also reducing the expression of proinflammatory cytokines (184).

NE, a transmitter released predominantly from the ends of sympathetic nerve fibers, can bind to two families of AR (alpha-(αAR) and beta-AR (βAR)) expressed on immune cells and affect immune responses under various conditions (185). For example, NE might induce anti-programmed cell death protein 1 (PD-1) monoclonal antibody resistance in lung adenocarcinoma via the inhibition of CD8^+^ T-cell infiltration and function. Activation of ARs in secondary lymphoid organs might cause the inhibition of interleukin-2 (IL-2) and then the inhibition of lymphocyte proliferation in adjuvant-induced arthritis (186). Moreover, once β2 AR is activated in Th0/Th1 cells, the increased cyclic adenosine monophosphate (cAMP) level can inhibit the production of interferon-gamma (IFN-γ) and promote the production of interleukin 4 (IL-4) production in Th2 cells, therefore promoting the humoral immunity and inhibiting cell-mediated immunity (187).

SP is produced in a subset of DRG neurons and, upon noxious stimulation, is released from peripheral and central (spinal) terminals (188). SP can regulate the activation and function (e.g., phagocytosis and cytokine secretion) and promote the cell survival/viability of some innate immune cells (e.g., natural killer cells, DCs, macrophages, neutrophils, mast cells, and eosinophils) (189). For example, SP/neurokinin 1 receptor (NK1R) signaling seems to activate two proinflammatory signaling pathways (protein kinase C (PKC) and phosphoinositide 3-kinases (PI3Ks)/protein kinase B (PKB)), leading to extracellular signal-regulated kinase 1/2 (ERK1/2)/NF-κB activation and cytokines/chemokine production in mouse macrophages (190). In addition, SP can stimulate mast cells to release proinflammatory factors such as C-C motif chemokine ligand 2 (CCL2), C-C motif chemokine ligand 3 (CCL3), C-C motif chemokine ligand 4 (CCL4), granulocyte-macrophage colony-stimulating factor (GM-CSF), interleukin 8 (IL-8), and TNF-α (188).

Second, neurotransmitters/neuropeptides (e.g., NE, ACh, SP, and VIP) from immune cells (182) can modulate PNS function by binding to specific receptors on neuron/nerve fibers and glial cells in PNS (**Figure 1A**) (180). These neurotransmitters/neuropeptides produced by immune cells might act through the paracrine pathway leading to nerve fiber/neurons/glial cells in PNS (or through an autocrine pathway to the immune cells themselves).

Third, cytokines/chemokines from the neuron/nerve fibers and glial cells of PNS (191, 192) can modulate immune cell function by binding to specific cytokine receptors on immune cells (**Figure 1B**) (20). For example, although enteric ganglion cells (EGCs) are non-immune cells, they directly sense invading pathogens via specific Toll-like receptors (TLRs) and release proinflammatory cytokines such as interleukin-1β (IL-1β) and IL-6 (123, 193). Rothan et al. have shown that primary neurons from human angiotensin-converting enzyme 2 (ACE2)-expressing mice produce cytokines (e.g., interferon-α (IFN-α), C-X-C motif chemokine ligand 10 (CXCL10), CCL2, IL-6, and TNF-α) after infection with severe acute respiratory syndrome coronavirus 2 (SARS−CoV−2) (194).

Lastly, cytokines/chemokines from the immune cells can modulate PNS function by binding specific receptors on neuron/nerve fibers and glial cells in PNS (**Figure 1B**) (182, 195). For example, sensory neurons (including nociceptors) can express receptors for cytokines, lipids, and growth factors (182, 196). Cytokines (e.g., TNF, IL-1β, IL-6, interleukin-17 (IL-17)) from macrophages, mast cells, and other immune cells interact with sensory neurons through these cytokine receptors during infection/inflammation, allergy, and tissue damage/injuries. In addition, bacteria such as *Staphylococcus aureus* can activate nociceptor sensory neurons that modulate pain and inflammation in the host (197).

**Figure 2:**
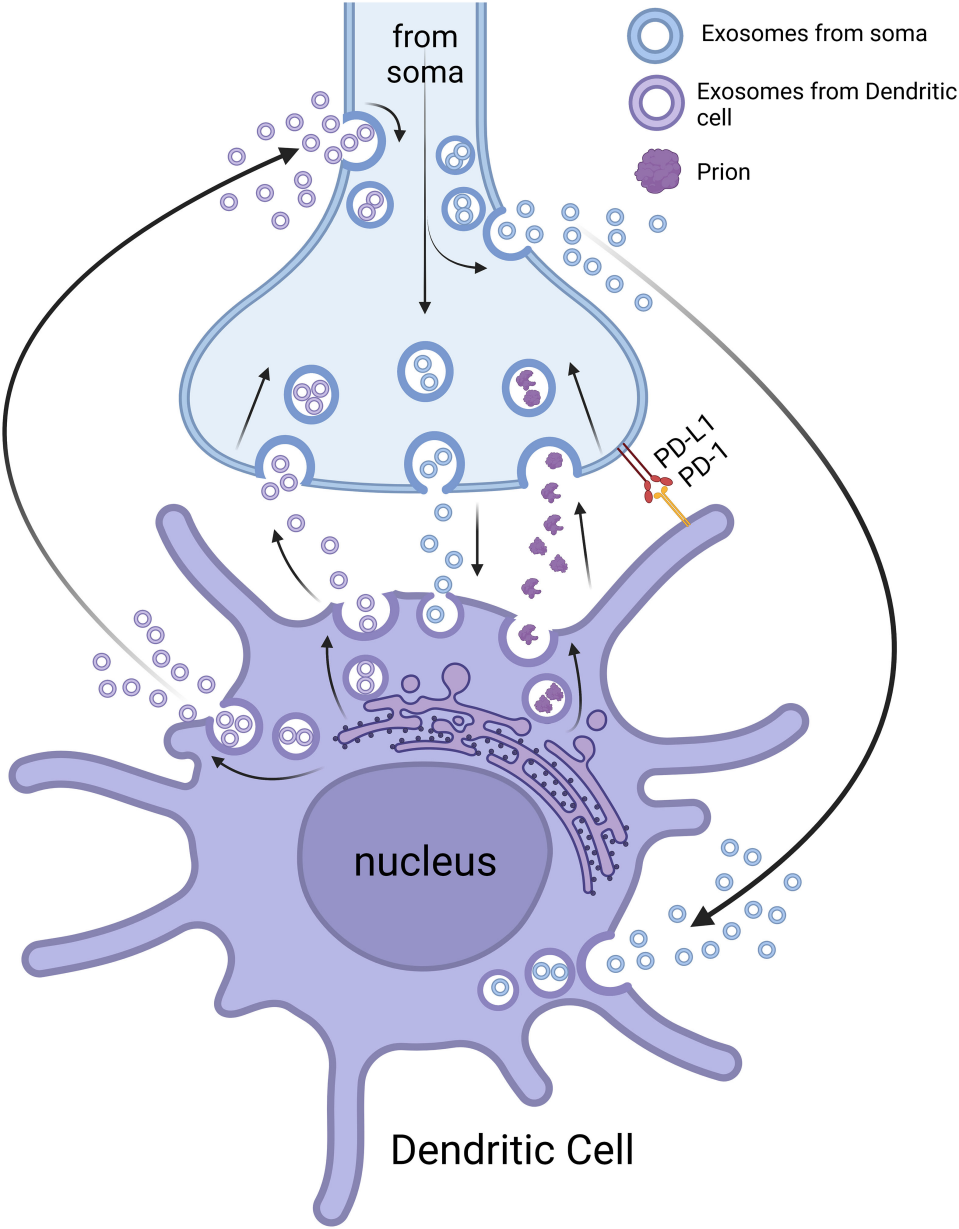
Neuroimmune crosstalk through extracellular vesicles and ligand-receptor pairs. NDEs can influence the function of immune cells (e.g., DCs) locally (neuroimmune cell units) and remotely. IEEs can also affect the function of nerve fibers/neurons locally (neuroimmune cell units) and remotely. The neuroimmune cell units can function as a potential route for prion transmission. The interaction of nerve and immune cells can also be through ligand-receptor pairs (e.g., PD-L1 and PD-1). Please note that the space (cleft) between the nerve terminals and immune cells is exaggerated for better presentation. The image was created by BioRender.

Another possible interaction between the nerve fibers and immune cells might occur through cell surface (insoluble) ligand-receptor pairs (36). Ligand-receptor pairs (**Figure 2**) are molecular interactions that take place between a specific signaling molecule called a ligand and a corresponding receptor protein. These interactions play a crucial role in cellular communication and signal transduction, allowing cells to respond to various stimuli (198). For example, programmed death-ligand 1 (PD-L1) on tumor cells can bind with PD-1 on T cells to reduce the proliferation of PD-1 positive cells (e.g., CD8^+^ cytotoxic T cells), inhibit their cytokine secretion, and induce apoptosis (199). Therefore, the PD-1/PD-L1 pathway represents an effective therapeutical target for immunotherapy of some cancers (199). Since most neuroimmune interaction occurs at discrete anatomical locations in which neurons and immune cells colocalize, the ligand-receptor interaction is crucial for the formation and maintenance of this type of cell-cell contact. For example, tumor-associated nerves can express PD-L1, and its level has been correlated with tumor-associated lymphocytes (e.g., CD8^+^ cytotoxic T cells) that might express PD-1 (200). Therefore, interaction through PD-1/PD-L1 or other cell surface ligand-receptor pairs might be a mechanism through which nerves and immune cells interact. However, further studies need to be carried out to confirm this type of interaction and to search for surface ligand-receptor pairs responsible for this neuroimmune interaction.

### 5.2 Neuroimmune crosstalk through extracellular vesicles

Extracellular vesicles (EVs) are lipid bilayer-delimited particles released by some cells into the extracellular space. They play essential roles in intercellular communication by transferring various molecules/substances between cells, including proteins, lipids, nucleic acids (RNA and DNA), and other bioactive molecules (201). Three main types of extracellular vesicles are known: exosomes, microvesicles (microparticles or ectosomes), and apoptotic bodies. These vesicles differ in their biogenesis, size, and cellular origins (201). EVs have been implicated in immune responses, tissue regeneration, cancer progression, and neurological disorders. Researchers are also exploring the potential of these vesicles as diagnostic biomarkers and therapeutic delivery vehicles because of their ability to traverse biological barriers and target specific cells (202).

Neuron-derived exosomes (NDEs) are probably released from damaged neurons/nerves/axons, as living cells are generally required for exosome production (203). NDEs can contain a variety of cargos, including proteins, lipids, RNA/microRNAs, and viruses, which can be transferred to recipient cells and influence their subsequent functions (204). By using a murine primary cortical neuron culture model, Zhou et al. have shown that Zika virus can be transmitted between neurons via NDEs (204). These exosomes might play a role in the propagation of signals between neurons, synaptic plasticity, and the regulation of neuronal development/function (203, 205, 206). NDE-mediated intercellular signaling might also contribute to a number of neurodegenerative diseases such as Alzheimer’s disease, Parkinson’s disease, and multiple sclerosis (206).

Immune cell exosomes (IEEs; immune cell-derived extracellular vesicles) released by various types of immune cells are crucial in immune regulation and intercellular communication within the immune system and beyond (207). They carry a diverse cargo of molecules that can modulate immune responses and influence the behavior of recipient cells. The different immune cell types, including DCs, macrophages, B cells, and T cells, release exosomes with distinct compositions and functions. IEEs can contain various bioactive molecules, such as proteins, lipids, cytokines/chemokines, and nucleic acids (207). These molecules can be transferred to target cells, such as other immune cells or non-immune cells (e.g., neuron and glial cells), to regulate immune responses or convey specific signals (**Figure 2**). For example, after forming immunological synapse with DCs, T cell-derived exosomes can promote antiviral responses of DCs (208). The study of IEEs has shown promise in various areas, including immunotherapy, vaccine development, and the treatment of certain autoimmune diseases (207, 209). For example, DC-derived exosomes can activate innate and adaptive immunity and therefore might have use as a vaccine with several advantages (e.g., good immunogenicity, delivery efficiency, application in the immunosuppressive environment) (210).

Recent studies have demonstrated that EVs have essential roles in neuroimmune crosstalk because of their ability to facilitate local and remote communication between cells and tissues (**Figure 2**) (211, 212). Observation of exosomes from nerves or immune cells at local contact points (e.g., by using EM or high-resolution light microscopic imaging) might be useful to confirm/understand the mechanism for this neuroimmune interaction. However, exosomes from projections/fragments of immune cell/nerve fibers might be difficult to discern in ultrathin sections by EM. If this is the case, exosome markers might prove useful for characterizing the exosomes from nerves and immune cells (213). Although some evidence exists for neuroimmune interactions through EVs, further studies should be performed to investigate the mechanisms of this interaction and to develop potential therapeutical approaches for the treatment of certain neurological/neuroimmunological and immunological/infectious diseases (214).

### 5.3 Anti-inflammatory reflex

The inflammatory reflex is a mechanism by which the sensory (afferent) signaling pathway is related to efferent-mediated output to regulate proinflammatory cytokine production and immune responses (215). The anti-inflammatory reflex (216) is a physiological mechanism that helps regulate and control/reduce the inflammation/immune responses in the human body. It involves neural pathways and signaling that actively suppress the production and release of proinflammatory molecules, thereby reducing inflammation (217). The primary pathway associated with the anti-inflammatory reflex is the cholinergic anti-inflammatory pathway, in which the vagus nerve is involved (216). The cholinergic anti-inflammatory pathway, namely the efferent or motor arm of the inflammatory reflex, might regulate innate immune responses against injury, pathogens, and tissue ischemia (218).

When injury/inflammation occurs, immune cells release proinflammatory molecules such as cytokines. These cytokines activate sensory nerve fibers that transmit signals to the brain via the vagus nerve. In response, the brain sends signals back through the vagus nerve to dampen the immune response and to reduce inflammation (219). Interestingly, activation/manipulation of the cholinergic anti-inflammatory pathway might be a novel therapeutic strategy against COVID-19 (220). When the anti-inflammatory pathway is activated, nerve terminals release ACh, which binds to ACRs on immune cells (primarily macrophages and T cells). The ACh pathways can then inhibit the production and release of proinflammatory cytokines, such as TNF-α and IL-1β, thereby exerting an anti-inflammatory effect. In our previous study, we have observed the expression of ACRs on DCs in mouse Peyer′s patches (36). The anti-inflammatory reflex is an essential mechanism for maintaining immune homeostasis and preventing excessive or prolonged inflammation. Dysregulation of this reflex has been implicated in various inflammatory diseases, such as sepsis, rheumatoid arthritis, and IBD (221–223).

Whereas some neuropeptides (e.g., NPY and SP) have proinflammatory effects, other neuropeptides (e.g., VIP, galanin, and opioid peptides) might be involved in the anti-inflammatory reflex (224–226). For example, in innate immunity, VIP seems to inhibit the production of inflammatory cytokines/chemokines from DCs, macrophages, and microglia (227). In adaptive immune response, VIP might reduce the proinflammatory Th1 and Th17 responses (228). In addition, opioid peptides released by immune cells can activate opioid receptors located on sensory nerve endings and therefore effectively reduce inflammatory pain (229).

### 5.4 Neuroimmune interactions as potential therapeutical targets

The modulation of neuroimmune interactions might lead to innovative interventions that modify disease progression or alleviate symptoms. The targeting of neuroimmune pathways offers a multifaceted approach for addressing complex conditions by influencing both immune responses and neural functions (230).

Acupuncture is a traditional Chinese medical practice that involves the insertion of thin needles into specific points on the body. These points, known as acupuncture points or acupoints, are believed to be located along pathways called meridians (231). Since neuroimmune crosstalk plays an essential role in the development and maintenance of inflammation and inflammatory pain, acupuncture (considered as mechanical/physical stimulation that stimulates the nociceptors and mechanical receptors in the skin, muscles, and other tissues) might thus reduce pain/inflammation and promote tissue repair/regeneration (232–234). For example, acupuncture suppresses serum levels of TNF-α/IL-6/IL-1β and improves animal survival in a murine model of endotoxemia (182). In addition, another study has shown that acupuncture possibly reduces the levels of several proinflammatory factors (e.g., neuropeptides, neurotrophins, and cytokines/chemokines) and disrupts the Th1/Th2 balance through the hypothalamic-pituitary-adrenal (HPA) axis pathway (235).

Vagus nerve stimulation (VNS), a United States Food and Drug Administration (FDA)-approved treatment for both drug-resistant depression and epilepsy, can produce clinically meaningful antidepressant and anti-seizure effects (11, 182, 236, 237). VNS can also be utilized for the treatment of other non-neurological diseases such as IBD and rheumatoid arthritis (133, 238). For example, VNS via an electric stimulator decreases the pain and inflammation in two-thirds of rheumatoid arthritis patients resistant to drugs such as methotrexate (239). VNS leads to sympathetic β2-AR signaling on T helper cells, which secrete ACh to activate splenic macrophages expressing nicotinic acetylcholine receptors (nAChRs) and then reduce the production of TNF-α and other proinflammatory cytokines (167). In addition, VNS can be performed by using ultrasound for site-selective neuromodulation in order to regulate specific physiological/pathophysiological functions (240).

VNS can also be utilized for the treatment of infectious diseases such as sepsis and COVID-19 (241–243). For example, transcutaneous auricular VNS can significantly inhibit the production of proinflammatory cytokines (e.g., TNF-α and IL-1β) and increase the production of anti-inflammatory cytokines (e.g., IL-4 and interleukin 10 (IL-10)) in sepsis patients (241). Severe cases of COVID-19 are characterized by excessive inflammatory responses (e.g., “cytokine storm”), and VNS is a possible treatment here, since it might reduce the levels of inflammatory markers (e.g., C-reactive protein and procalcitonin) (243).

In addition to physical stimulation, chemical/pharmaceutical manipulations of neuroimmune interaction can also be used in the development of treatment/therapy for certain diseases/disorders. By using this approach, PNS is manipulated in order to treat certain inflammatory/infectious diseases. For example, rheumatoid arthritis is a chronic autoimmune disease with chronic inflammation/imbalanced ANS; restoration of the ANS balance might represent an innovative/effective treatment for rheumatoid arthritis (244). In another study, NGF therapy seemed to improve bone marrow sensory innervation, to increase blood cell production, and then to reduce the occurrence of peripheral ischemia. Therefore, nociceptors might provide a new target for treating ischemic complications in diabetes (245). In addition, by using a mouse infection model, another study has shown that CGRP can inhibit the recruitment of neutrophils/opsonophagocytic killing of *Streptococcus pyogenes* and blocking CGRP signaling to immune cells might be utilized for the treatment of this skin infection (246).

Intra-tumoral innervation refers to the nerves/nerve fibers within or around tumor tissues/tumor TLT (247). Nerves can infiltrate tumors and establish connections with cancer cells/immune cells/stromal cells, creating a neural network within the tumor microenvironment. Tumor innervation can influence various aspects of cancer biology, including tumor growth/progression, angiogenesis, immune response, and metastasis (247). Tumor nerve-derived transmitters/neuropeptides can promote tumor cell proliferation, survival, and migration, while also affecting tumor-associated inflammation and immune cell infiltration. Understanding the complex interactions between nerves and tumors might provide insights into novel therapeutic strategies targeting tumor innervation to modulate cancer progression and to improve treatment outcomes (248, 249). For example, selective α2- and β2-AR agonists/antagonists have been used in the treatment of experimental models of autoimmune diseases, fibromyalgia, and chronic fatigue syndrome (249).

Manipulation of the immune system may also be utilized to treat PNS-associated diseases (e.g., peripheral neuropathy) (225, 229, 250). Peripheral neuropathy, as a result of damage/injury to peripheral nerves, often causes weakness/numbness/pain (usually in the hands and feet) (251). It can also affect other areas and body functions (e.g., digestion, urination, and circulation). For example, inhibition of osteoclast activity by alendronate can modify the aberrant subchondral bone remodeling and reduce the innervation/pain during the early stage of osteoarthritis (252). In addition, modulation of the macrophage phenotype might benefit peripheral nerve repair/regeneration (253, 254). Furthermore, galanin, a biologically active neuropeptide widely distributed in the CNS/PNS and the endocrine system, has been shown to have analgesic (pain-relieving) effects, particularly in the context of inflammatory pain conditions (255). Although the exact mechanisms by which galanin reduces inflammatory pain are not fully understood, it appears to modulate pain perception through its interactions with various receptor systems (e.g., galanin receptor 1 and galanin receptor 2) and its ability to influence the release of neurotransmitters (e.g., ACh, NE, serotonin, and dopamine) (256–259). Immunotherapy has recently been tested for its potential applications in the treatment of immune-mediated peripheral neuropathies such as Guillain-Barré syndrome, chronic inflammatory demyelinating polyradiculoneuropathy, and neuropathy associated with IgM anti-myelin-associated glycoprotein (260).

Of note, although neuroimmune interactions have been explored as potential therapeutic targets, many of these approaches are still in the experimental stages, and their applications have not been established (230). Rigorous clinical trials must be performed to determine their safety and efficacy, and regulatory approval, such as FDA clearance, must be obtained before they can be considered established treatments (236, 237).

## 6 Conclusion

Studies investigating neuroimmune interactions and modulation will significantly contribute to a better understanding of the mechanisms through which the PNS potentially affects cellular and humoral-mediated immune responses or vice versa in health and diseases. Neuroimmune cell units provide an anatomical and physiological basis for bidirectional crosstalk between the PNS and the immune system in peripheral tissues, including lymphoid tissues and organs. Furthermore, physical, chemical, pharmacological, and other manipulations of these neuroimmune interactions should bring about the development of practical therapeutic applications for certain neurological, neuroimmunological, infectious, inflammatory, and immunological disorders/diseases (239, 261, 262).

## Author contributions

AB: Writing – original draft. DH: Writing – original draft. MJA: Writing – review & editing. PKN: Writing – review & editing. WKG: Conceptualization, Project administration, Writing – review & editing. BM: Conceptualization, Project administration, Supervision, Writing – review & editing.
